# Impaired fat oxidation during exercise in multiple acyl‐CoA dehydrogenase deficiency

**DOI:** 10.1002/jmd2.12024

**Published:** 2019-03-14

**Authors:** Karen L. Madsen, Nicolai Preisler, Astrid E. Buch, Mads G. Stemmerik, Pascal Laforêt, John Vissing

**Affiliations:** ^1^ Copenhagen Neuromuscular Center, Department of Neurology Copenhagen Neuromuscular Center, Rigshospitalet Copenhagen Denmark; ^2^ Neuromuscular Center, Department of Neurology, Neuromuscular Center Raymond‐Poincaré Hospital Garches France; ^3^ INSERM U1179, END‐ICAP Versailles Saint‐Quentin‐en‐Yvelines University Montigny‐le‐Bretonneux France

**Keywords:** exercise, fatty acid oxidation disorders, inherited metabolic disease, multiple acyl‐CoA dehydrogenase deficiency, muscle disease, stable isotopes

## Abstract

We investigated the in vivo skeletal muscle metabolism in patients with multiple acyl‐CoA dehydrogenase deficiency (MADD) during exercise, and the effect of a glucose infusion. Two adults with MADD on riboflavin and l‐carnitine treatment and 10 healthy controls performed an incremental exercise test measuring maximal oxidative capacity (VO_2max_) and a submaximal exercise test (≤1 hour) on a cycle ergometer. During submaximal exercise, we studied fat and carbohydrate oxidation, using stable isotope tracer methodology and indirect calorimetry. On another day, the patients repeated the submaximal exercise receiving a 10% glucose infusion. The patients had a lower VO_2max_ than controls and stopped the submaximal exercise test at 51 and 58 minutes due to muscle pain and exhaustion. The exercise‐induced increase in total fatty acid oxidation was blunted in the patients (7.1 and 1.1 vs 12 ± 4 μmol × kg^−1^ × min^−1^ in the healthy controls), but total carbohydrate oxidation was higher (67 and 63 vs 25 ± 11 μmol × kg^−1^ × min^−1^ in controls). With glucose infusion, muscle pain decreased and average heart rate during exercise dropped in both patients from 124 to 119 bpm and 138 to 119 bpm. We demonstrate that exercise intolerance in MADD‐patients relates to an inability to increase fat oxidation appropriately during exercise, which is compensated partially by an increase in carbohydrate metabolism.

## 1 INTRODUCTION

Multiple acyl‐CoA dehydrogenase deficiency (MADD, OMIM#231680) is an autosomal recessively inherited lipid storage myopathy. Patients with neonatal MADD suffer severe metabolic decompensation often with a lethal outcome, while the late‐onset disease has a milder course dominated by symptoms of muscle weakness, exercise intolerance, and episodes of hypoglycemia.^1^, ^2^

MADD is caused by defects in electron transporters electron transfer flavoprotein (ETF) or electron transfer flavoprotein ubiquinone oxydoreductase (ETF‐QO). Acyl‐CoA dehydrogenases initiate the beta‐oxidation of fatty acids in the mitochondria. They pass electrons to ETF and ETF‐QO that transfer them on to the respiratory chain. Defective flavoproteins therefore restrict the function of the acyl‐CoA dehydrogenases and reduce flux through beta‐oxidation.^3^

Most patients with *ETFDH* mutations respond to riboflavin treatment. Riboflavin is a precursor of flavin adenine dinucleotide (FAD), an essential cofactor for the flavoproteins.^3^, ^4^ Riboflavin improves muscle function and has been reported to completely reverse symptoms in some patients.^5^ Carnitine is a cofactor for fatty acid oxidation. Carnitine conjugated to long‐chain fatty acids facilitates their transport into the mitochondria for oxidation and ensures the removal unoxidized fatty acids. Many MADD patients are also treated with l‐carnitine to compensate for the carnitine that is washed out with unoxidized fatty acids. The clinical effect of this is unknown, but likely diminishes the toxic effect of acyl‐carnitines on sarcolemmal integrity.^6^ Riboflavin and l‐carnitine thus alleviate muscle symptoms and prevent acute metabolic decompensation, but many patients still complain of muscle pain and fatigability during exercise.^1^

Despite extensive descriptions of the clinical phenotypes related to this rare disease, the impact of MADD on skeletal muscle metabolism in vivo is poorly understood. We studied whole‐body metabolism and exercise performance in MADD patients and the effect of IV‐glucose supplementation.

## 2 METHODS

### 2.1 Subjects

We studied 2 women with late‐onset MADD on riboflavin and l‐carnitine treatment and 10 healthy sedentary age‐matched controls (Table 1). Six of the healthy controls were included in parallel for this and another study using the same exercise protocol since some of the patients exercised at identical intensities as in this study.^7^ The four other healthy controls were included prospectively for this study protocol. Patient 1 is compound heterozygous for mutations in the *ETFDH* gene: c.622G>C in exon 6 and c.1241_1246del in exon 10.^1^ Patient 1 had complained of episodic muscle pain since her twenties and the mutation was identified when she was 47 years of age. Patient 2 has a novel homozygous c.1194G>C mutation in the *ETFDH* gene, which was identified in her and other siblings when she was a young child after her brother was admitted to the hospital with severe hypoglycemia. Both have a history of exercise intolerance and report improvement with riboflavin treatment (duration >10 years) and patient 1 had taken coenzyme Q10 for <5 years, which she reported had led to additional improvement. Both have near normal muscle strength, except mild proximal lower limbs weakness (MRC 4). Patient 2 had habitually elevated plasma‐creatine kinase (250‐2500 U/L) and patient 1 had experienced one episode of elevated creatine kinase.

**Table 1:** Demographics and maximal exercise test results in 2 patients with multiple acyl‐CoA dehydrogenase deficiency and 10 healthy controls

	Patient 1	Patient 2	Healthy controls	
			Mean ± SD	Range
*Demographics*				
Age (years)	50	20	32 ± 14	18‐65
Gender (female:male)	Female	Female	7:3	‐
BMI (kg/m^2^)	24	33	24 ± 3	19‐28
l‐carnitine dose (g/day)	3	2	‐	‐
Riboflavin dose (mg/day)	100	100	‐	‐
Coenzyme Q10 dose (mg/day)	250	‐	‐	‐
*Maximal exercise tests*				
VO_2max_ (mL × kg^−1^ × min^−1^)	26	17	43 ± 6	30‐50
Heart rate_max_ (bpm)	152	187	189 ± 12	166‐202
Workload_max_ (Watt)	80	105	255 ± 35	215‐330
Blood‐lactate_max_ (mmol/L)	5.4	6.1	8.8 ± 2.4	6.1‐13.5
Blood‐glucose_rest_ (mmol/L)	5.7	4.6	5.4 ± 0.2	4.2‐6.6
Blood‐glucose_max_ (mmol/L)	5.3	4.1	6.1* ± 0.7	5.2‐7.2
Plasma‐creatine kinase_rest_ (U/L)	74	1470	92 ± 0.7	55‐126

BMI, Body Mass Index; bpm, beats per minute; max, value at maximal exercise intensity; SD, standard deviation; submax, mean value of submaximal exercise period; VO_2max_, maximal oxygen uptake.

* *P* < 0.05 vs rest.

All subjects gave written informed consent prior to study procedures and the study was conducted in accordance with the Declaration of Helsinki. The study is registered at www.clinicaltrials.gov, identifier: NCT02635269.

### 2.2 Maximal exercise test

Maximal oxygen uptake (VO_2max_) was determined by an incremental exercise test on a cycle ergometer (Lode Excalibur Sport 925900, Lode BV, Groningen, the Netherlands). Gas exchange was measured with a metabolic cart (CPET, Cosmed Srl., Milan, Italy). Blood glucose and lactate, and plasma creatine kinase levels were measured.

### 2.3 Submaximal exercise—Substrate turnover

After a day of rest, all subjects performed a submaximal exercise test until exhaustion or maximally for 1 hour after 4 to 12 hours of fasting. The workload was constant and was set to match 60% of VO_2max_ for the patients (patient 1: 35 W, patient 2: 40 W). The healthy controls exercised at the same absolute workloads as the patients (37 ± 8 W) to match energy expenditure.

During the submaximal exercise, total fatty acid and carbohydrate oxidation rates were studied with indirect calorimetry and calculated using the nonprotein respiratory quotient.^8^ We used a constant infusion of stable isotope tracers to measure the palmitate rate of oxidation (U‐^13^C‐palmitate) and the turnover of glycerol (1,1,2,3,3‐D5‐glycerol). Blood samples were drawn every 10 minutes through a venous catheter in a hand vein and analyzed for metabolites, hormones and stable isotope tracers. Simultaneously, breath samples were collected for analysis of metabolized tracer as ^13^CO_2_ (see the “Supplemental Methods” for more details). Plasma acyl‐carnitines were measured as previously described.^9^

### 2.4 Submaximal exercise—Glucose infusion

At least 2 days after the first submaximal test, the patients repeated the submaximal exercise test without the tracers. Instead, they received intravenous glucose 10% as a bolus of 2 mL × kg^−1^ 10 minutes before exercise and a continuous infusion of 4.7 mL × kg^−1^ × h^−1^ during exercise. The healthy controls did not perform the submaximal exercise with glucose infusion.

### 2.5 Statistics

Results for the healthy controls are reported as mean ± SD. Differences between rest and exercise were assessed with a paired Student's *t*‐tests. *P* < 0.05 was considered significant.

## 3 RESULTS

### 3.1 Maximal exercise

Patients reached VO_2max_ and maximal workloads that were half that of the healthy controls. Blood‐glucose dropped in both patients, while it increased in all the healthy controls (Table 1).

### 3.2 Submaximal exercise—Substrate turnover

**Figure 1:**
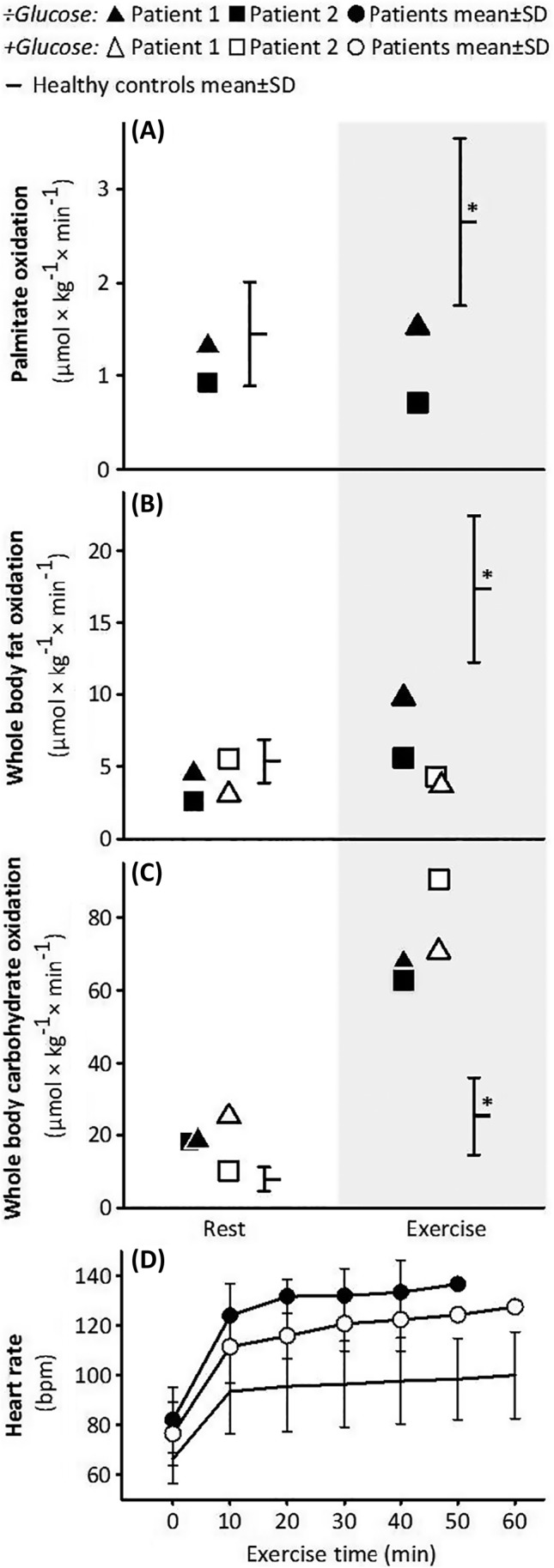
Fat and carbohydrate oxidation and heart rate (panel D) during submaximal exercise in two adults with multiple acyl‐CoA dehydrogenase with (+) and without (÷) a 10% glucose infusion and in a group of 10 healthy controls. Panel (A) palmitate rate of oxidation was measured with stable isotope technique, and (B) and whole‐body fat oxidation and (C) whole‐body carbohydrate oxidation were measured with breath‐by‐breath indirect calorimetry. *P < 0.05 vs rest

Patient 1 exercised for 58 minutes and Patient 2 for 51 minutes. Oxygen consumption was 944 ± 20 and 1113 ± 63 mL × kg^−1^ × min^−1^ during exercise, respectively, which was similar to the healthy controls (947 ± 139 mL × kg^−1^ × min^−1^). Both complained of pain in thighs and calves, which was the reason for stopping exercise before 60 minutes. The relative workload was higher in patients, as reflected by their higher heart rates during exercise (Figure 1), and average rates of perceived exertion (Borg scale) of 15 ± 3 and 16 ± 4 vs 9 ± 1 in healthy controls (Table 2).

**Table 2:** Metabolites during submaximal exercise in 2 patients with multiple acyl‐CoA dehydrogenase deficiency and 10 healthy controls

	Substrate turnover				Glucose infusion				Substrate turnover	
	Patient 1		Patient 2		Patient 1		Patient 2		Healthy controls	
	Rest	Exercise	Rest	Exercise	Rest	Exercise	Rest	Exercise	Rest	Exercise
*Metabolites*										
Free fatty acids (μmol/L)	536	999 ± 255	1414	1736 ± 189	467	113 ± 83	638	507 ± 109	643 ± 258	798 ± 410*
Plasma‐palmitate (μmol/L)	289	359 ± 71	197	185 ± 13	‐	‐	‐	‐	173 ± 38	213 ± 39*
R_a_ gly (μmol × min^−1^ × kg^−1^)	5.0	9.0 ± 0.5	7.4	9.7 ± 1.5	‐	‐	‐	‐	4.3 ± 1.0	6.9 ± 1.7*
Blood‐lactate (mmol/L)	0.6	1.3 ± 0.3	0.9	5.1 ± 0.5	0.6	1.7 ± 0.3	1.3	3.3 ± 1.3	0.8 ± 0.1	0.7 ± 0.1*
Blood‐glucose (mmol/L)	5.1	4.9 ± 0.2	4.4	4.1 ± 0.2	12.5	8.8 ± 1.5	11.7	10.7 ± 1.4	5.3 ± 0.5	5.4 ± 0.6

Mean values of the exercise period are reported ± SD, standard deviation; R_a_ gly, rate of appearance glycerol.

* *P* < 0.05 vs rest.

#### 3.2.1 Substrate turnover

Both patients produced lactate and their blood glucose dropped, while lactate and glucose remained stable in the healthy controls (Table 2). Palmitate oxidation during exercise was lower in the patients than in the healthy controls. It increased in patient 1 while it dropped in patient 2 during exercise. Whole‐body fat oxidation was less than half of the healthy controls during exercise. Conversely, whole‐body carbohydrate oxidation increased threefold in the patients during exercise and was twice the value of the controls (Figure 1).

Lipolysis was seemingly unaffected as indicated by an increase in the release of free fatty acids and glycerol in response to exercise in all subjects. Insulin levels dropped with exercise in both patients in the same manner as in the controls and the catecholamines increased similarly in patients and healthy controls (Table Se‐1).

### 3.3 Submaximal exercise—Glucose infusion

Heart rate during exercise was lower in both patients with glucose (Figure 1). Rate of perceived exertion also dropped by 1.5 points on the Borg scale on average, and the patients reported less pain during exercise on glucose. The whole‐body fat oxidation (Figure 1) remained at resting values during exercise in both patients. Total carbohydrate oxidation increased in the patients and the glucose was consumed as plasma values dropped during the infusion and exercise (Table 2).

### 3.4 Acyl‐carnitine profiles

*Patient 1*: Had a normal‐to‐high plasma concentration of free carnitine and discretely increased medium chain acyl‐carnitines at rest. After 60 minutes of exercise, the medium‐chain acyl‐carnitines increased along with a few short‐chain acyl‐carnitines. With IV‐glucose, the acyl‐carnitines after exercise were nearly all within normal range.

*Patient 2*: Had a normal concentration of free carnitine in plasma and markedly increased concentrations of acyl‐carnitines of all lengths. The concentration of acyl‐carnitines was the same after exercise and did not change with the glucose infusion.

## 4 DISCUSSION

This case study shows that patients with MADD on riboflavin and l‐carnitine treatment are unable to increase fat oxidation appropriately during exercise, leading to a severe impairment of skeletal muscle energy metabolism compromising exercise performance. Limited fat metabolism, but no exercise intolerance, has been suggested in a previous study of two mildly affected children performing low‐intensity exercise.^10^ Our study shows this directly by stable isotope technique and by pushing fat oxidation even further by using submaximal exercise, which revealed symptoms of exercise intolerance. This was despite treatment with riboflavin and l‐carnitine. Both have normal free carnitine concentrations with the l‐carnitine treatment, but we saw a correlation between degree of acyl‐carnitine accumulation and the impairment in fatty acid oxidation. Patient 1 increased fatty acid oxidation in response to exercise, her plasma‐CK value was within reference range and she only has discrete accumulation of medium‐chain acyl‐carnitines. Patient 2 cannot increase fat oxidation from resting level, her plasma‐CK was multiple times above upper reference and she had markedly increased plasma concentrations of acyl‐carnitines of all lengths.

Patient 2 had a high insulin level at rest after 4 hours of fasting (Table 2), which is indicative of a prediabetic condition. However, with the onset of exercise, the translocation of GLUT 4 and muscular glucose uptake is largely stimulated by the muscle contractions in an insulin‐independent manner.^11^, ^12^ Insulin drops and the catecholamines increase response to exercise in patient 2 as it does in patient 1 and in the healthy controls and her total carbohydrate oxidation, glucose consumption and lactate production were comparable to that of patient 1.

Furthermore, our patients had very low maximal workload and oxidative capacities and the relatively high lactate levels observed at the end of submaximal exercise probably reflect the impairment of mitochondrial function due to the abnormal electron transfer to respiratory chain complexes. During submaximal exercise, carbohydrate oxidation was exaggerated to fuel the working muscle in our patients, and blood glucose dropped with exercise indicating that gluconeogenesis and glycogenolysis in liver and muscle are struggling to keep up with the demand for extramuscular glucose as fuel. In healthy individuals performing 1‐hour moderate intensity exercise, endogenous energy supplies form fat and glycogen can keep up with energy demand, and glucose supplementation makes no difference on exercise performance.^13^, ^14^, ^15^

IV‐glucose alleviated leg pain and improved exercise tolerance in our patients. The same effect is found in patients with other inborn errors of lipid metabolism.^16^ Whether this effect can be obtained by ingesting sucrose before exercise needs to be determined, as IV‐glucose, but not oral sucrose, improves exercise capacity in carnitine palmitoyl transferase II deficiency.^16^

## CONFLICTS OF INTEREST

Karen L. Madsen reports having received travel and research support from Reata Pharmaceuticals; Nicolai Preisler reports having received research support and honoraria from Sanofi/Genzyme; Pascal Laforêt reports having received research and travel support, and/or speaker honoraria from Sanofi/Genzyme and served as consultant on advisory boards for Sanofi/Genzyme; John Vissing reports having received research and travel support, and/or speaker honoraria from Sanofi/Genzyme, Ultragenyx Pharmaceuticals, Santhera Pharmaceuticals, and aTyr Pharma, and served as consultant on advisory boards for Sanofi/Genzyme, aTyr Pharma, Ultragenyx Pharmaceuticals, Santhera Pharmaceuticals, Sarepta Therapeutics, Audentes Therapeutics, NOVO Nordisk, Alexion Pharmaceuticals, and Stealth Biotherapeutics within the last 3 years. Astrid E. Buch and Mads G. Stemmerik have no conflicts of interest to report.

## AUTHOR CONTRIBUTIONS

*Karen L. Madsen*: Acquisition of data, analysis and interpretation of data, study supervision, study concept and design, and drafting of the manuscript.

*Nicolai Preisler*: Acquisition of data, analysis and interpretation of data, study supervision, study concept and design, and critical revision of manuscript for intellectual content.

*Astrid E. Buch*: Acquisition of data, analysis and interpretation of data, and critical revision of manuscript for intellectual content.

*Mads G. Stemmerik*: Acquisition of data, analysis and interpretation of data, and critical revision of manuscript for intellectual content.

*Pascal Laforêt*: Study supervision, study concept and design, and critical revision of manuscript for intellectual content.

*John Vissing*: Analysis and interpretation of data, study supervision, study concept and design, and critical revision of the manuscript for intellectual content.

## ETHICS APPROVAL

The study was approved by the IRB of the Capital Region of Denmark.

## PATIENT CONSENT

All subjects gave written informed consent prior to study procedures and the study was conducted in accordance with the principles of the Declaration of Helsinki.

## DATA AND MATERIAL AVAILABILITY

The corresponding author has full access to all data which is filed within Rigshospitalet, the Capital Region of Denmark in accordance with the Danish Data Protections Laws and the principles of Good Clinical Practice. The corresponding author has the right to publish any and all data separate and apart from any sponsor.

## Supporting information


**Supplemental Data 1** MADD_Supplemental methods

**Figure S1** Plasma carnitine and acyl‐carnitine concentrations in two patients with multiple acyl‐CoA dehydrogenase deficiency at rest and after exercise without and with IV‐glucose (+ glucose). C4‐C18: Acylcarnitines of 4‐18‐carbon lengths.

**Table S1** Hormones at rest and end‐exercise, during submaximal exercise in 2 patients with multiple acyl‐CoA dehydrogenase deficiency and 10 healthy controls
