# Supplementary material for: Impaired fat oxidation during exercise in multiple acyl‐CoA dehydrogenase deficiency
Source: JIMD Rep. 2019 Mar 14;46(1):79–84. doi: 10.1002/jmd2.12024 (PMC6498824; doi:10.1002/jmd2.12024)
Supplement: Supplementary file 1 — Supplemental Data 1 MADD_Supplemental methods [file JMD2-46-79-s001.docx]

SUPPLEMENTAL METHODS:

**Substrate turnover study:**

When the patients arrived at the laboratory, we collected baseline breath and blood samples. We injected priming doses of NaH13CO3 (85.0µg×kg-1) and 1,1,2,3,3-D5-Glycerol (73.5µg×kg-1) (Cambridge Isotope laboratories Inc., Andover, MA, USA). We started an infusion of U-13C-palmitate (2.6µg×kg-1×min-1) and 1,1,2,3,3-D5-Glycerol (4.9µg×kg-1×min-1) dissolved in 500mL 5% human albumin. The infusion was given with an IMED Gemini PC-2-pump (ALARIS Medical Systems, Inc., CA, USA) and started two hours before exercise to obtain steady state and the infusion rate was doubled with the onset of exercise. We collected exhaled air (Douglas bag, Hans Rudolph Inc., KS, USA) every 10 minutes, transferred it to vacutainer tubes and analyzed it for the enrichment of ^13^CO_2_. At the same time points, blood samples were drawn through a venous catheter in a hand vein. The hand was kept warm with a heating pad in order to arterialize the sampled blood. Stable isotope tracer rates of appearance were calculated with Steeles non-steady-state equation for stable isotopes (Steele 1959). Palmitate rate of oxidation was calculated as described by van Loon et al. (van Loon et al. 2003) Indirect calorimetry calculations were performed using a non-protein respiratory quotient described by Peronnet et al. (Péronnet and Massicotte 1991).

**Blood and breath samples**:
Blood was drawn for the analysis of for lactate and glucose in heparinized syringes and immediately analysed with ABL-90, 725 system (Radiometer, Copenhagen, Denmark). For the analysis of creatine kinase, blood was collected on tubes coated with lithium heparin and analyzed with an enzymatic assay and spectrophotometry at the dept. of clinical biochemistry at Rigshospitalet.
Blood for all other analyses was collected in cooled EDTA-tubes (0.33M, 10µL×mL^-1^) and spun (Centrifuge 5702R, Eppendorf AG, Hamburg, Germany) at 4000 rpm for 10 minutes at 4ºC. Plasma was transferred to cooled Eppendorf tubes which were frozen on dry ice and stored at -80ºC until analysis. Plasma was sent for analysis of insulin at the dept. of clinical biochemistry, Rigshospitalet (Cobas 8000, Roche, Rotkreuz Switzerland). Plasma free fatty acid and epinephrine and nor-epinephrine were analyzed by spectrophotometry (Multiskan GO, Thermo Scientific, SkanIt™ Software, Thermo Fisher Scientific Inc., USA).

Stable isotopes ^13^CO_2_ in breath and palmitate enrichment in plasma were analyzed with gas chromatography-combustion-isotope ratio mass spectrometry (Finnigan MAT, Bremen, Germany). Glycerol in plasma was analyzed with liquid chromatography-tandem mass spectrometry (LC-MS/MS).

REFERENCES

Péronnet F, Massicotte D (1991) Péronnet F, Massicotte DTable of nonprotein respiratory quotient: an update. Can J Sport Sci 16:23-29. Can J Sport Sci J Can Sci Sport 16:23–9

Steele R (1959) Influences of Glucose Loading and of Injected Insulin on Hepatic Glucose Output*. Ann N Y Acad Sci 82:420–430. doi: 10.1111/j.1749-6632.1959.tb44923.x

van Loon LJ, Koopman R, Schrauwen P, et al (2003) The use of the [1,2-13C]acetate recovery factor in metabolic research. EurJApplPhysiol 89:377–383. doi: 10.1007/s00421-003-0810-x
