# Supplementary figures and images for: Impaired fat oxidation during exercise in multiple acyl‐CoA dehydrogenase deficiency
Source: JIMD Rep. 2019 Mar 14;46(1):79–84. doi: 10.1002/jmd2.12024 (PMC6498824; doi:10.1002/jmd2.12024)

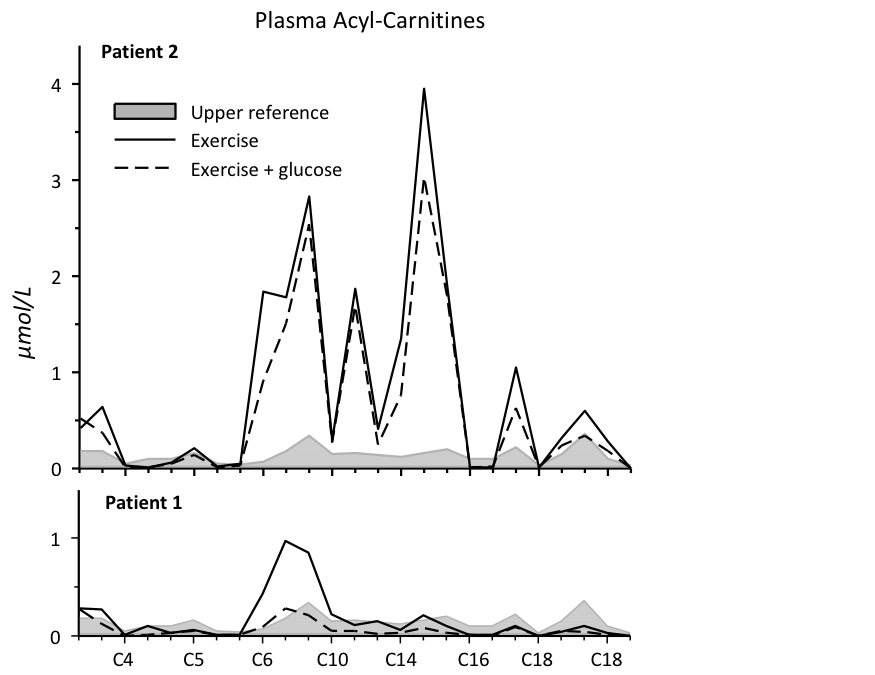

Supplement: Supplementary file 2 — Figure S1 Plasma carnitine and acyl‐carnitine concentrations in two patients with multiple acyl‐CoA dehydrogenase deficiency at rest and after exercise without and with IV‐glucose (+ glucose). C4‐C18: Acylcarnitines of 4‐18‐carbon lengths. [file JMD2-46-79-s002.png]
