# Supplementary material for: Impaired fat oxidation during exercise in multiple acyl‐CoA dehydrogenase deficiency
Source: JIMD Rep. 2019 Mar 14;46(1):79–84. doi: 10.1002/jmd2.12024 (PMC6498824; doi:10.1002/jmd2.12024)
Supplement: Supplementary file 3 — Table S1 Hormones at rest and end‐exercise, during submaximal exercise in 2 patients with multiple acyl‐CoA dehydrogenase deficiency and 10 healthy controls [file JMD2-46-79-s003.docx]

**Table e-1**. Hormones at rest and end-exercise, during submaximal exercise in two patients with Multiple Acyl-CoA Dehydrogenase deficiency and 10 healthy controls

|  | Substrate turnover | | | | | | Glucose infusion | | | | | | **Healthy controls** | | | |
| --- | --- | --- | --- | --- | --- | --- | --- | --- | --- | --- | --- | --- | --- | --- | --- | --- |
|  | **Patient 1** | | | **Patient 2** | | | **Patient 1** | | | **Patient 2** | | |  |  |  |  |
|  | *Rest* | ***Exercise*** | | *Rest* | ***Exercise*** | | *Rest* | ***Exercise*** | | *Rest* | ***Exercise*** | | *Rest* | | ***Exercise*** | |
| ***Hormones*** |  |  |  |  |  |  |  |  |  |  |  |  |  |  |  |  |
| Insulin *(µmol/L)* | 28 | **19** | | 222 | **102** | | 25 | **72** | | 250 | **1015** | | 36 | ±20 | **30** | ±16 |
| Epinephrine *(pg/mL)* | 106 | **269** | | 103 | **334** | | 36 | **209** | | 30 | **84** | | 194 | ±187 | **290** | ±27 |
| Nor-epinephrine *(ng/mL)* | 1.1 | **1.5** | | 0.6 | **1.7** | | 0.7 | **1.9** | | 0.4 | **0.8** | | 3.7 | ±0.2 | **3.7** | ±0.1 |

Mean values are reported *±* SD: Standard Deviation
